# Nutrition of preterm infants in relation to bronchopulmonary dysplasia

**DOI:** 10.1186/1471-2466-11-7

**Published:** 2011-02-03

**Authors:** Andreas Wemhöner, Daniel Ortner, Edda Tschirch, Alexander Strasak, Mario Rüdiger

**Affiliations:** 1 Medical University Innsbruck, Department for Pediatrics, Neonatology; Austria; 2 Technical University Dresden; University Hospital Dresden, Department for Pediatric Intensive Care and Neonatology, Germany; 3 Medical University Innsbruck, Department of Medical Statistics, Informatics and Health Economics; Austria

## Abstract

**Background**

The pathogenesis of bronchopulmonary dysplasia (BPD) is multifactorial. In addition to prenatal inflammation, postnatal malnutrition also affects lung development.

**Methods**

A retrospective study was performed to analyse during the first two weeks of life the total, enteral and parenteral nutrition of premature infants (<31 weeks, birth weight ≤1500 g) born between 08/04 and 12/06.

**Results**

Ninety-five premature infants were analysed: 26 with BPD (27 ± 1 weeks) and 69 without BPD (28 ± 1 weeks). There was no statistical significant difference in the total intake of fluids, calories, glucose or protein and weight gain per day in both groups. The risk of developing BPD was slightly increased in infants with cumulative caloric intake below the minimal requirement of 1230 kcal/kg and a cumulative protein intake below 43.5 g/kg. Furthermore, the risk of developing BPD was significantly higher when infants had a cumulative fluid intake above the recommended 1840 ml/kg. In infants who developed BPD, the enteral nutrition was significantly lower than in non-BPD infants [456 ml/kg (IQR 744, 235) vs. 685 (IQR 987, 511)]. Infants who did not develop BPD reached 50% of total enteral feeding significantly faster [9.6 days vs. 11.5].

**Conclusions**

Preterm infants developing BPD received less enteral feeding, even though it was well compensated by the parenteral nutrient supply. Data suggest that a critical minimal amount of enteral feeding is required to prevent development of BPD; however, a large prospective clinical study is needed to prove this assumption.

## Background

Bronchopulmonary dysplasia (BPD) is a chronic pulmonary disease that affects preterm infants. In the past, BPD was mainly caused by ventilatory injury and affected about 30% of preterm infants with birth weights below 1000 grams [1]. Gentler ventilation techniques, antenatal steroids and surfactant treatment have reduced the incidence of lung injury. Despite this progress, the incidence of BPD is not decreasing, but the picture of BPD is changing. The so called "new BPD" is a lung development problem that involves impairment of alveolarisation which results in large, simplified alveolar structures, dysmorphic capillary configuration and variable interstitial cellularity and/or fibroproliferation [2].

Several factors are considered responsible for altering lung development and may subsequently support the development of BPD. In addition to prenatal inflammation, nutrition plays an important role in normal lung development and maturation [2,3] Nutrition has a direct effect on the developing lung because it can modulate lung structure. General under-nutrition in humans leads to lung emphysema [4,5]. In rats, caloric restriction reduces the alveolar number by 55% and the alveolar surface area by 25% [6]. Seventy two hours after re-feeding, however, rat lungs are re-modelled with normal alveolar numbers and surface areas [7].

Sufficient nutrition is often difficult to achieve in preterm infants. Due to various problems associated with immaturity, extremely preterm infants receive only minimal enteral nutrition during the first weeks of life and require supplemental parenteral nutrition. However, there is an ongoing debate concerning the required amount of protein, carbohydrates and calories [8]. A sufficient amount of protein and calories seems to be necessary for organ growth; thus, a low protein or caloric intake could impair lung development, resulting in BPD.

The present study was performed to test the hypothesis that nutrition intake during the first 2 weeks of life is lower in infants who subsequently develop BPD. Furthermore, the effects of protein, calorie and carbohydrate intake and the percentage of enteral nutrition during the first two weeks on the subsequent development of BPD were studied. The charts for all preterm infants under 31 weeks and below 1500 g who were born in our unit during a 17-month period were analysed.

## Methods

The observational cohort study was performed in the neonatal intensive care unit (NICU) of a tertiary centre at the Department of Pediatrics, Neonatology at the University Hospital Innsbruck, Austria. The medical ethics committee of the Medical University Innsbruck approved the study.

### Data collection

Data were obtained from all preterm infants born between August 2004 and December 2006. All infants with birth weights below 1500 g and gestational ages below 31 weeks who were treated in the NICU during that period were included in the study. Infants were excluded from the analysis for the following reasons: death within the first week of life, transfer to another hospital and missing data.

The following demographic variables were obtained for all preterm infants born during the study period: birth weight, gestational age, mode of delivery, singleton pregnancy, 5 minute APGAR score and completed courses of antenatal steroids given.

The main outcome parameters were the cumulative amount of protein, calorie, carbohydrate and enteral feeding intake at 14 days of life. Parameters of total nutrition were obtained daily, and parameters of neonatal growth (weight, head circumference and length) were obtained daily up to day 14 and were then obtained at day 28 and 36 weeks postmenstrual age.

The following morbidity parameters were collected: Respiratory Distress Syndrome (RDS) incidence, surfactant application after intubation, surfactant application and the presence of an open ductus arteriosus as determined by echocardiography.

The following discharge parameters were obtained: body weight and length, head circumference and length of stay in hospital.

### Definition of bronchopulmonary dysplasia

The aim of the study was to compare nutritional data during the first 14 days of life in infants who later developed BPD with data of non-BPD infants. BPD was defined as treatment with oxygen >21% for at least 28 days on 36 week postmenstrual age or discharge to home, whichever comes first as described elsewhere [9].

### Enteral nutritional regime

During the study period, the feeding regime was not changed. Preterm infants were fed according to a local protocol that was adapted by the attending neonatologist to meet the actual requirements of the individual patient [10,11]. According to NICU routine, oral feeding is started with either 0.5 [birth weight (b.w.) ≤ 750 g] or 1 ml (b.w. > 751 g) mother milk or donor breast milk as soon as possible after birth. Subsequently, the daily feeding volume was increased by about 10 ml/kg body weight. The volume was neither increased nor decreased if the attending staff noticed "feeding intolerance". Preterm infants were either fed with their mother's milk or donor breast milk. Milk fortification was usually performed if a feeding volume of more than 100 ml/kg*d was achieved.

### Parenteral nutritional regime

Enteral nutrition was supplemented with parenteral nutrition if needed. Actual requirements were calculated daily with software that calculates fluid, calories, protein and glucose requirements for enteral and parenteral nutrition and lipids for parenteral application. The required parenteral nutrition was gradually tapered as enteral feeding volumes increased and was generally stopped if a feeding volume of 130 to 140 ml/kg of fortified milk was achieved [12]. The protocol for parenteral nutrition started with a total of 70-80 ml/kg/d fluid on the first day of life [13]. The fluid intake was increased by 10-20 ml/kg/day until 150 ml/kg/d was achieved. Protein administration was started during the first day of life with 1 g/kg/day and was increased by 0.5 g/kg per day until 3 g/kg is achieved. On the third day, intravenous lipid administration was started with 0.5 to 1 g/kg/d and was advanced in increments of 0.5 to 1 g/kg/d until 3 g/kg/d was achieved.

It is assumed that a "minimal nutritional requirement" is needed for undisturbed growth and that problems occur only below that critical threshold. Therefore, minimal requirements during the first two weeks were calculated according to recent recommendations [13,14] protein 43.5 g/kg, calories 1110-1210 kcal/kg and carbohydrates 187-213 g/kg. Because volume overload is associated with the subsequent development of BPD, a "maximal" total fluid intake during the first two weeks of life was calculated at 1620-1800 ml/kg. The relative risk of developing BPD was calculated if the minimal requirements were not achieved.

### Data analysis

Continuous data, which is normally distributed, is presented as mean/SD; for skewed data, medians with interquartile ranges are shown. Statistical analyses of outcome were conducted in order to compare data for BPD and non-BPD infants. Univariate comparisons of proteins, calories, carbohydrates and total fluid were performed using the non-parametric Mann-Whitney U-Test. All reported p-values are two-sided with a level of significance set at 0.05. All analyses were performed using SPSS 15.0 (Chicago, Illinois).

## Results

### Perinatal data of the study group

All together, 100 infants were born and treated during the study period. 5 infants were excluded from the analysis because of death (n = 2) within the first week of life or transfer to another hospital (n = 3). The incidence of BPD in the study population was 27%. The perinatal data for BPD and non-BPD infants are shown in table 1. As expected, infants who developed BPD had a lower gestational age and birth weight (p < 0.02). These infants also had lower average weight, length and head circumference at the 14^th ^day of life and at a postmenstrual age of 36 weeks (p < 0.02 table 2) but the weight gain per day was not different.

**Table 1:** Perinatal data Perinatal data for infants in BPD and non-BPD (NBPD) group.

	BPD	NBPD
Patients (number)	26	69

Gestational age (weeks)	27 ± 1*	28 ± 1

Birth weight (gram)	965 ± 255*	1170 ± 246

Length (cm)	35 ± 3.2*	37 ± 2.5

Head circumference (cm)	24.7 ± 2.1*	26 ± 1.6

Apgar score at 5 minutes	7.8 ± 1	7.9 ± 1

Female infants	12 (46%)	38 (55%)

Singletons	16 (61%)	50 (72%)

Caesarean section	25 (96%)	67 (97%)

Small for gestational age	3 (11%)	5 (7%)

Completed courses of antenatal steroids	25 (96%)	65 (94%)

*p < 0.02 vs. NBPD		

Data are mean ± standard deviation or number and percentage (in parentheses)

**Table 2:** Body composition Data for the study population at the 14th day of life and at 36 weeks post-conception.

	Day 14		36 Weeks	
	**BPD**	**NBPD**	**BPD**	**NBPD**

Weight (grams)	1015 ± 254*	1190 ± 241	1883 ± 286*	2038 ± 286

Length (cm)	36.8 ± 3.3*	38.5 ± 2.6	42 ± 2*	43 ± 2

Head circumference (cm)	25 ± 2*	26.7 ± 1.7	30 ± 1.5*	31.4 ± 1.5

*p < 0.01 vs. NBPD				

Data are mean ± standard deviation

### Cumulative nutrition management

**Figure 1:**
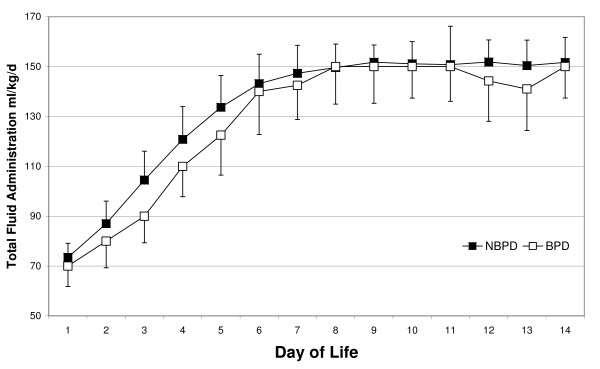
**Amount of daily fluid intake during the first two weeks of life**. Data are the means and standard deviations for infants from the NBPD (open square) and BPD (black square) groups.

The difference in total fluid intake during the first two weeks was not statistically significant between infants with and without subsequent BPD (table 3). As shown in figure 1, the increase in fluid administration was similar in both groups. However, all infants who received more than the "maximal total fluid intake" of 1840 ml/kg developed BPD (relative risk in comparison to infants without BPD: p = < 0.01 RR 4.00, 95% CI 2.6, 6.2).

**Table 3:** Nutrient intake Total nutrient intake during the first two weeks of life.

	BPD	NBPD
Total fluid [ml/kg]	1864 (1792, 1929)	1860 (1813, 1904)

Total calories [kcal/kg]	1089 (966, 1190)	1154 (1081, 1221)

Total protein [g/kg]	46 (41, 49)	45 (41, 49)

Total carbohydrates [g/kg]	100 (187, 103)	102 (96, 108)

Data are median and interquartile range (in parentheses)

The difference in the total caloric intake during the first two weeks of life was not statistically significant between infants with and without subsequent BPD (table 3). The risk of developing BPD was slightly (but not significantly) higher if the caloric intake during the first two weeks of life was below a minimal requirement of 1230 kcal/kg (RR 1.92, 95% CI 0.6, 5.7).

The total intake of amino acids during the first two weeks of life was not significantly different between the two groups (table 3); however, there were large variations between infants. Again, the risk of developing BPD was (not significantly) higher if the protein intake was below the minimal requirement of 43.5 g/kg (RR 1.2, 95% CI 0.6, 2.4). Finally, the intake of carbohydrates during the first two weeks of life was not significantly different between the two groups (table 3).

### Enteral nutrition

**Figure 2:**
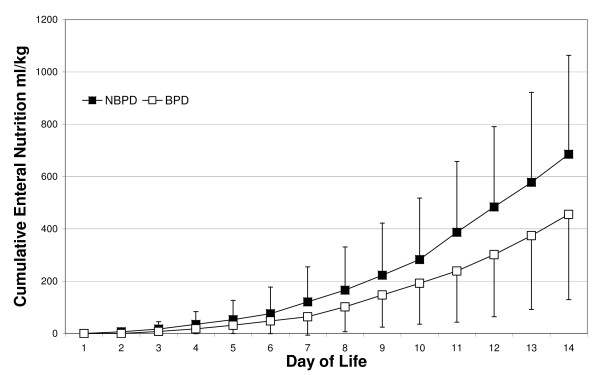
**Cumulative amount of enteral feeding during the first two weeks of life**. Data are the means and standard deviations for infants from the NBPD (open square) and BPD groups (black square).

**Figure 3:**
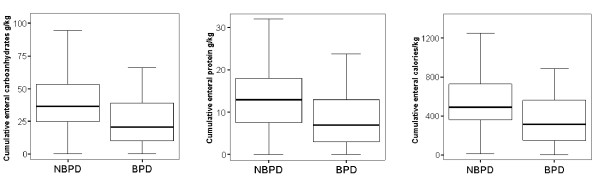
**Cumulative enteral management during the first two weeks of life is shown for the NBPD and BPD groups**. Cumulative enteral carbohydrates, protein and calories (* p < 0.01, vs. control).

As shown in figure 2, the two groups differed with respect to the amount of enteral feeding. Infants who did not develop BPD received significantly more enteral feeding during the first two weeks of life when compared with infants who developed BPD (p < 0.04) (Figure 3a). Whereas 50% of enteral feeding was achieved at a median of 9.6 days in infants who did not develop BPD, a median of 11.5 days was required to achieve this level of feeding in infants who developed BPD (p < 0.01). In sum, the enteral intake of carbohydrates (figure 3a), proteins (figure 3b) and calories (figure 3c) during the first two weeks of life was lower (p < 0.01) in infants who developed BPD.

### Clinical outcome and other aspects of preterm morbidity

Analysis of data on postnatal respiratory morbidity revealed that significantly more infants received surfactant in the BPD group (80 vs. 52%, p < 0.01), whereas the incidence of RDS was similar in both groups (100 vs. 94%). There was a higher rate of ROP in the BPD group (57 vs. 21%, p = 0.001) and IVH (30 vs. 7% p = 0.004). Furthermore, the incidence of a persistent ductus arteriosus (42%), need for treatment ductus arteriosus (38%) and NEC > stage II (0%) were the same in both groups.

Finally, the length of stay in the hospital was significantly longer for infants with BPD than for infants who did not develop BPD (96 vs. 60 days, p < 0.01), respectively.

## Discussion

The present study tested the hypothesis that infants who develop BPD have lower caloric or protein intake during the first two weeks of life than infants without BPD. However, data from the current study do not support this assumption; the total intake of fluids, calories, amino acids, carbohydrates and weight gain per day was similar in infants with and without BPD. However, infants who developed BPD had a significantly lower amount of enteral nutrition during the first two weeks of life. Furthermore, the present study supports previous reports of the importance of fluid restriction during the first weeks of life to prevent the development of BPD [15,16]. Whereas the median fluid intake was similar in both groups of the present study, infants who received more than the recommended amount of fluid had a significantly higher risk of developing BPD. In contrast to other studies [17,18] we did not find any significant differences in calories and fat intake in the first 14 days of life. The reasons therefore might be a greater number of patients and a longer observational period in those studies.

Data from the present study are of great clinical interest because the impact of nutrition on BPD development has been discussed for a long time. *Sosenko et al *hypothesized that general under-nutrition, specifically insufficient protein intake, may increase the vulnerability of a preterm infant to oxidant-induced lung injury and the development of "old" BPD [19]. Furthermore, nutrition plays an important role in lung growth and development. The "new BPD" is characterised by a rarefaction of alveolar structures, which could be partially explained by insufficient protein or caloric intake during the postnatal period. According to the present study, though, not all infants who developed BPD had low caloric intake. If intake of calories or protein was below the minimal requirement, infants were more likely to develop BPD. However, the values did not reach statistical significance due to the small number of patients in this study.

One of the major findings of the present study is the lower enteral intake during the first two weeks in infants who developed BPD. Infants with a greater illness severity may be at a higher risk of BPD, and may also have a greater magnitude of feeding intolerance or a reluctance on the part of the clinician to aggressively increase enteral feeds. It could be assumed that parenteral nutrition alone is insufficient to meet the nutritional needs of preterm infants [20,21]. Furthermore, enteral nutrition could also have some beneficial effects for lung development. However, previous studies on enteral nutrition did not find a similar association between low enteral intake and subsequent development of BPD [22]. While this study is of clinical interest, some limitations should be discussed. First of all, the data were obtained retrospectively. It was the aim of the study to show whether infants who develop BPD received fewer calories and protein despite the very strict guidelines on nutrition in our neonatal intensive care unit. Thus, it was of interest to see a large variation between infants. A second limitation is the small number of infants, which may have caused type II errors. Over a long study period of more than two years, changes in management are likely and, thus, interpretation of the data would be more difficult. However, the present study provides a sound base for a prospective study on this subject.

## Conclusions

In conclusion, the present study shows that infants who developed BPD received less enteral feeding during the first two weeks of life, which was well compensated by the parenteral nutrient supply. It seems that a critical amount of protein and caloric intake is required to prevent the development of BPD; however, a large prospective randomized trial is needed to prove this assumption.

## Competing interests

The authors declare that they have no competing interests.

## Authors' contributions

AW had primary responsibility for protocol development, patient screening, enrolment, outcome assessment, preliminary data analysis and writing the manuscript. DO was responsible for patient screening. ET participated in the development of the protocol and analytical framework for the study and contributed to the writing of the manuscript. AS performed the final data analyses. MR supervised the design and execution of the study and contributed to the writing of the manuscript. All authors read and approved the final manuscript

## Pre-publication history

The pre-publication history for this paper can be accessed here:

http://www.biomedcentral.com/1471-2466/11/7/prepub
